# Understanding Resistance Mechanisms to Trifluralin in an Arkansas Palmer Amaranth Population

**DOI:** 10.3390/genes12081225

**Published:** 2021-08-10

**Authors:** Fidel González-Torralva, Jason K. Norsworthy

**Affiliations:** Department of Crop, Soil, and Environmental Sciences, University of Arkansas, Fayetteville, AR 72704, USA; jnorswor@uark.edu

**Keywords:** GST, herbicide resistance, P450, Palmer amaranth, resistance mechanism, trifluralin

## Abstract

*Amaranthus palmeri* S. Watson (Palmer amaranth) is considered a problematic and troublesome weed species in many crops in the USA, partly because of its ability to evolve resistance to herbicides. In this study, we explored the mechanism of resistance in a trifluralin-resistant *A. palmeri* accession collected from Arkansas, USA. Dose-response assays using agar plates demonstrated an *EC*_50_ (effective concentration that reduces root length by 50%) of 1.02 µM trifluralin compared to 0.39 µM obtained in the susceptible accession. Thus, under these conditions, the resistant accession required 2.6 times more trifluralin to inhibit root length by 50%. Seeds in the presence or absence of the cytochrome P450-inhibitior malathion displayed a differential response with no significant influence on root length, suggesting that resistance is not P450-mediated. In addition, application of 4-chloro-7-nitrobenzofurazan (NBD-Cl), a glutathione *S*-transferase (GST) inhibitor, showed significant differences in root length, indicating that GSTs are most likely involved in the resistance mechanism. Sequencing of *α*- and *β*-*tubulin* genes revealed no single nucleotide polymorphisms (SNPs) previously described between accessions. In addition, relative gene copy number of *α*- and *β*-*tubulin* genes were estimated; however, both resistant and susceptible accessions displayed similar gene copy numbers. Overall, our results revealed that GST-mediated metabolism contributes to trifluralin resistance in this *A. palmeri* accession from Arkansas.

## 1. Introduction

*A. palmeri*, a dicotyledonous species native to southwestern USA, is one of the most troublesome weeds of agronomic crops in the USA [1,2]. Different herbicides have been used for its control, and overreliance has gradually led to the appearance of *A. palmeri*-resistant accessions [3]. *A. palmeri* accessions with multiple resistance to herbicides have frequently been reported. For instance, accessions resistant to imazethapyr [4], trifluralin [5], dicamba [6], atrazine [7], glyphosate [8], fomesafen [9], *S*-metolachlor [10], and mesotrione [11] have been documented.

Trifluralin (*α*, *α*, *α* -trifluoro-2,6-dinitro-*N*,*N*-dipropyl-*p*-toluidine), was first commercially released in 1964 to control grass and dicotyledonous weeds using preplant soil incorporated applications in different row cropping systems [12]. During 2018, in *Glycine max* (L.) Merr. alone, more than 500,000 kg of trifluralin were used in the USA [13]. Trifluralin is a member of the dinitroaniline herbicide family, inhibiting microtubule (formed by the *α*- and *β*-*tubulin* heterodimers) assembly/polymerization. In eukaryotes, microtubules are cytoskeletal elements required for intracellular transport and cell division [14]. Inhibition of microtubule assembly/polymerization causes a mitosis alteration that triggers growth interruption and finally plant death [15,16]. Root swelling and stunting are the main symptoms developed by plants germinating in trifluralin-treated soil [16,17]. In *Zea mays* L. seedlings, radial elongation of cortical cells and multinucleate cells in meristematic tissues have been observed. In addition, suppression in the synthesis of DNA, RNA and protein have been reported in the presence of trifluralin [18].

Overreliance of herbicides, together with no alternatives in weed management strategies, has resulted in the appearance of herbicide-resistant accessions. Resistance to trifluralin has been reported mostly in grass weed species, a few of which include *Eleusine indica* (L.) Gaertn., *Setaria viridis* (L.) P. Beauv., and *Lolium rigidum* Gaudin [15,19,20,21]. These weeds have overcome herbicide effects through both target and nontarget site resistance mechanisms. Target-site resistance mechanisms can involve an amino acid change mainly in the *α*-*tubulin* gene at different positions. Known mutations in this gene that confer resistance include Leu-125-Met, Leu-136-Phe, Val-202-Phe, Thr-239-Ile, Arg-243-Met/Lys and Met-268-Thr [15,20,22,23,24]. Conversely, trifluralin-resistant weed accessions have evolved the ability to convert herbicide to nontoxic forms (less toxic polar conjugates) avoiding plant death or damage [17,25]. Other studies have demonstrated that trifluralin metabolic resistance can be reversed when the resistant accession is subjected to the herbicide prosulfocarb, a very long-chain fatty acid elongase-inhibiting herbicide, or by using phorate, a cytochrome P450 metabolism-inhibiting organophosphate insecticide [17,26].

Although resistance to trifluralin in *A. palmeri* has been reported previously [5], the resistance mechanism/(s) involved have not been described. In this paper, we used physical (dose-response assays), physiological (inhibition of metabolism), and molecular (*α*- and *β*-*tubulin* gene sequencing and gene amplification) approaches to describe the resistance mechanisms in a trifluralin-resistant *A. palmeri* accession collected in Arkansas, USA.

## 2. Materials and Methods

### 2.1. Plant Material

The resistant and susceptible accessions used in this study were documented in earlier experiments described elsewhere [10,27]. In order to characterize the resistance to trifluralin, the resistant accession was further evaluated. Previous experiments demonstrated that agar plates are a useful tool for selecting seedlings with suspected herbicide resistance [10]. Seeds of the putative-resistant accession were sown and processed as described below in the dose-response experiments section. At 10 days after treatment (DAT), seedlings with no trifluralin symptoms (e.g., swollen stem, necrosis) and long roots compared to the susceptible accession were transplanted into pots containing growing medium (Premier Horticulture Inc., Quakertown, PA, USA) and placed under greenhouse conditions at a 35/25 °C day/night temperature regime and a 16-h photoperiod until flowering. Harvested seeds from these plants were used for all experiments described below.

### 2.2. Dose-Response Experiments on Agar Plates

Fifteen seeds of resistant and susceptible accessions were placed in agar square plates (100 mm × 100 mm × 15 mm; Simport Scientific, Beloeil, QC, Canada) to compare side by side. Autoclaved agar plates contained 0.22% (*w*/*v*) of Murashige and Skoog Basal Salt Mixture (M524, Phytotechlab, Lenexa, KS, USA) and 1.1% (*w*/*v*) agar (Sigma Aldrich, St Louis, MO, USA). Agar solution pH was adjusted to 5.8 with HCl. Square plates contained 40 mL agar media plus different trifluralin concentrations. Trifluralin concentrations were 0, 0.0625, 0.125, 0.25, 0.5, 1.0, 2.0, 4.0, 8.0, and 16.0 µM. Agar plates were then placed vertically for 48 h at 28 °C under dark conditions and then moved to the greenhouse under the conditions described above. At 10 DAT, agar plates were photographed, and root lengths measured using ImageJ software [28]. Data were pooled across runs as no interactions were detected (*p* ≥ 0.05). Data were subjected to a four-parameter logistic regression model [29]:

$$Y=min+\left\{(max-min)∕\left[1+{\left(x/EC_{50}\right)}^{Hillslope}\right]\right\}$$

where *Y* corresponds to root length of seedlings; *min* and *max* represent the lower and upper asymptotes, respectively; *Hillslope* represents the slope of the curve; the *EC*_50_ corresponds to herbicide rate that inhibits the root length by 50%, and *x* represents the herbicide rate (µM). Regression analysis was conducted using SigmaPlot v.14.0 software (Systat Software Inc., San Jose, CA, USA). The resistance factor (RF) was obtained by dividing the *EC*_50_ of the resistant accession by the *EC*_50_ of the susceptible accession. The experiment was triplicated with one treatment plate per concentration per run, and root lengths were expressed in cm.

### 2.3. Metabolism Assays

Seeds of resistant and susceptible *A. palmeri* accessions were placed on agar plates containing either malathion, a P450-inhibiting insecticide, or NBD-Cl, a GST-inhibitor, to determine if metabolic resistance is involved in the resistant accession. Pilot experiments demonstrated that 8 µM malathion did not affect root length compared to water-treated plates. NBD-Cl treatments were set up at 50 nM, since under our lab conditions it was suitable for GST inhibition [10]. Treatments included a nontreated control (water), malathion (8 µM) or NBD-Cl (50 nM), trifluralin (1 µM; concentration derived from dose-response assays) and trifluralin + malathion (1 µM + 8 µM) or trifluralin + NBD-Cl (1 µM + 50 nM). Then, agar plates were managed as described in the previous section, and root length was measured accordingly. Each treatment consisted of fifteen seeds per accession with two accessions per plate per run, and the experiment was repeated twice. Data obtained were subjected to analysis of variance using Statistix software (Analytical Software, Tallahassee, FL, USA), and means were separated using the Tukey test at a 95% confidence level.

### 2.4. Tubulin Gene Sequencing

Approximately 50 mg of young fresh tissue was collected from resistant and susceptible plants and immediately frozen in liquid nitrogen for total RNA extraction. Total RNA was extracted using the Monarch Total RNA Miniprep Kit (New England Biolabs, Ipswich, MA, USA). RNA quantification was performed spectrophotometrically using a nanodrop (Nanodrop 2000c, Thermo Scientific, Waltham, MA, USA). Complementary DNA (cDNA) was synthesized with 1 µg of total RNA as template using the iScript Reverse Transcription Supermix (Bio-Rad Laboratories Inc., Hercules, CA, USA) in a 20 µL reaction. Primer sets were designed to amplify the region where mutations have been reported previously for trifluralin resistance [22,23,24,30]. Primers were designed using the Primer3Plus (available at https://primer3plus.com/cgi-bin/dev/primer3plus.cgi (accessed on 5 November 2020)) software [31]. The forward (5′-TCCACATTGGTCAAGCAGGT-3′) and reverse (5′-ATCTGCACCCTCAGCTCCTA-3′) primers were used to amplify 1304 bp of the *α*-*tubulin* gene. In addition, the forward (5′-CTGAAGGTGCCGAGTTGATTG-3′) and reverse (5′-ACCAATGCAAGAAAGCCTTCC-3′) were used to amplify 873 bp of the *β*-*tubulin* gene. Polymerase Chain Reactions (PCRs) were performed using 1 µL cDNA (50 ng), 1× Colorless GoTaq Flexi Buffer (Promega Corp., Madison, WI, USA), 1.5 mM MgCl_2_, 0.2 mM dNTP’s, 0.2 µM each forward and reverse primer, and 0.625 units GoTaq^®^ Hot Start Polymerase (Promega Corp., Madison, WI, USA) in a 25 µL reaction volume. Cycling conditions for the *α*-*tubulin* gene were as follows: 94 °C for 2 min; 40 cycles at 94 °C for 30 s; 65 °C for 30 s, 72 °C for 75 s, then a final cycle at 72 °C for 5 min. Cycling conditions for the *β*-*tubulin* gene were similar except for the extension cycle being adjusted to 72 °C for 50 s. Assessment of correct amplification was carried out by loading 5 µL of PCR products onto a 1.2% agarose gel. PCR products were then purified using the Wizard SV Gel and PCR Clean up System (Promega Corp., Madison, WI, USA). At least three biological replicates per accession were sequenced in both senses (Eurofins Genomics, Louisville, KY, USA).

### 2.5. Tubulin Gene Copy Number Estimation

Approximately 100 mg of leaf tissue per biological replicate was collected and stored at −80 °C until genomic DNA (gDNA) extraction. gDNA was extracted from leaf tissue using the E.Z.N.A. Plant DNA kit (Omega Bio-Tek Inc., Norcross, GA, USA) following the manufacturer’s directions. gDNA was quantified spectrophotometrically (Nanodrop 2000c, Thermo Scientific, Waltham, MA, USA) and diluted to 10 ng µL^−1^ using deionized water. gDNA was used to estimate the relative *α* and *β*-*tubulin* gene copy number in resistant and susceptible accessions by quantitative PCR (qPCR) following the MIQE guidelines [32]. Homologs of the *Cinnamoyl*-*CoA reductase* (*CCR*) and *peter Pan*-*like* (*PPAN*) genes in *A. palmeri* were used as reference genes, as they are present as a single gene copy in the Arabidopsis genome and *Lolium* species [33,34,35]. Primers were designed as described in the previous section and amplicon length in all primer sets was between 100–130 bp (Table 1). To assess target specificity, dissociation curves were generated at the end of each run to ensure that no unspecific product was amplified. Standard curves for each primer set were generated with gDNA dilutions ranging from 1.56 to 25 ng and percentage efficiency (E) calculated by E = [10^(−1/slope)^ − 1] × 100 [36] (Table 1). Each 20 µL qPCR reaction contained 10 µL of 2× SsoAdvanced Universal SYBR Green Supermix (Bio-Rad Laboratories Inc., Hercules, CA, USA), 0.8 µL of each forward and reverse primer at 10 µM, and 25 ng gDNA. All qPCR reactions were run on 96-well plates on a CFX Connect Real-Time System (Bio-Rad Laboratories Inc., Hercules, CA, USA). Thermal cycling was as follows: 98 °C for 3 min, 40 cycles of 98 °C 10 s, and 61 °C for 30 s. Dissociation curves were created by increasing the temperature from 65 °C to 95 °C, 0.5 °C every 5 s. On each qPCR plate, no template controls were included for each primer set, where gDNA was replaced by water. Threshold cycles (Ct) were calculated using CFX Maestro software (Bio-Rad Laboratories Inc., Hercules, CA, USA) and relative gene copy number calculated using modification of the 2^−ΔΔCt^ method and expressed as 2^ΔCt^ [37,38,39]. Results were used to generate the fold increase in *α* and *β*-*tubulin* relative to *CCR* and *PPAN*. Experiments had four biological replicates per accession, each of them run with four technical replicates for each gene. Data obtained were averaged and analyzed using Student’s *t*-test to detect differences between accessions.

## 3. Results and Discussion

### 3.1. Dose-Response Experiments on Agar Plates

**Figure 1:**
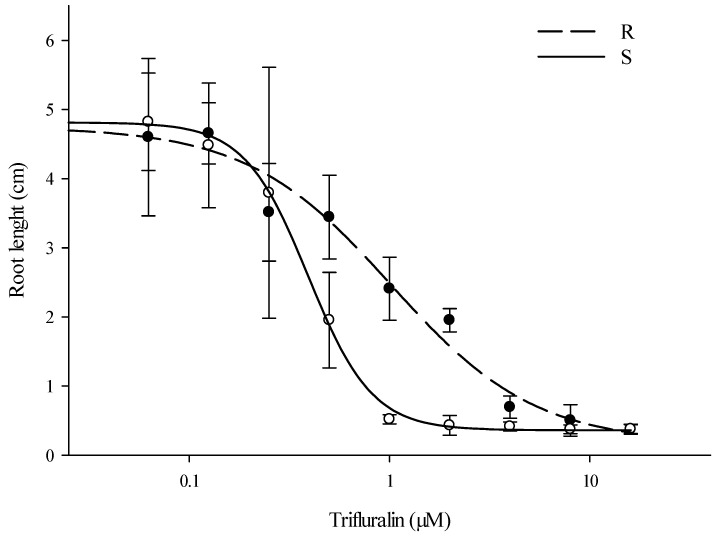
Dose-response curves of *A. palmeri* seedlings treated with trifluralin herbicide. Root lengths were obtained at 10 days after treatment. Data were subjected to a four-parameter logistic curve. R: resistant accession; S: susceptible accession. Vertical bars represent ± standard deviations of the mean.

Root length in both accessions decreased as the trifluralin concentration increased, and at 10 DAT visual differences were clearly observed between accessions. At this time, using 1 µM trifluralin, the seedlings from the resistant accession were healthier and had longer roots compared to susceptible seedlings. In addition, susceptible seedlings displayed swollen stems, which is a typical symptom caused by trifluralin. Using agar plates, the dose-response curves showed an *EC*_50_ of 1.02 and 0.39 µM for the resistant and susceptible accession, respectively. Thus, under these conditions the resistant accession required 2.6 times more herbicide than the susceptible accession to have a 50% root reduction (Figure 1). Under field conditions, Gossett et al. [5] reported that five to six times more herbicide was needed to control a trifluralin-resistant *A. palmeri* based on visual and shoot dry weights. In entire plant dose-response experiments, an Australian trifluralin-resistant *L. rigidum* accession was six times more resistant than the wild type used for comparison [22]. In *Setaria italica* L. Beauv. using a rapid-seed bioassay on glass petri dishes with different tubulin-inhibiting herbicides, resistant seedlings showed approximately eight-fold resistance to trifluralin [20].

### 3.2. Metabolism Assays

**Figure 2:**
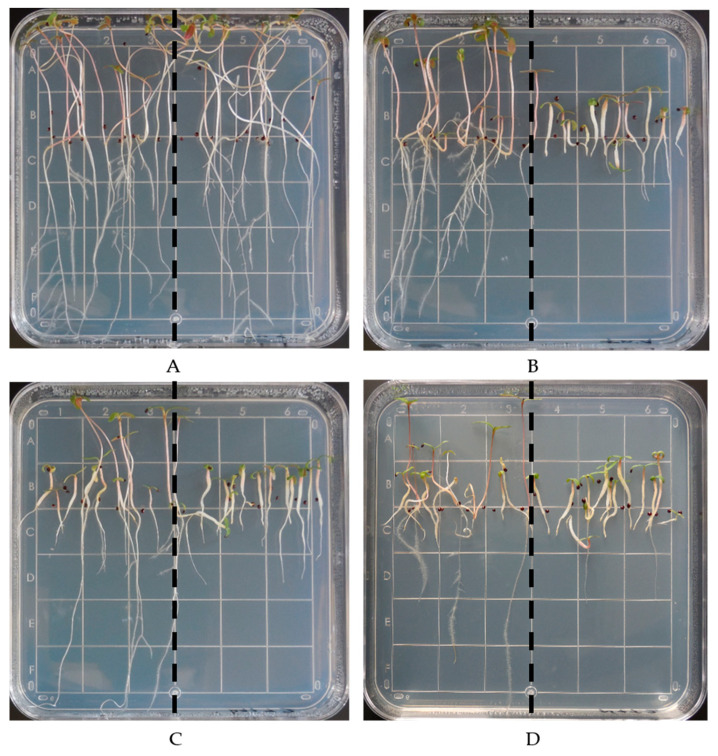
Representative agar plates showing the response of *A. palmeri* seedlings under different treatments. Control (**A**); trifluralin 1 µM (**B**); trifluralin 1 µM + malathion 8 µM (**C**); and trifluralin 1 µM + NBD-Cl 50 nM (**D**). In all plates, left and right sides correspond to resistant and susceptible accession seedlings, respectively. Photographs were taken at 10 days after treatment.

Seeds of trifluralin-resistant and susceptible *A. palmeri* accessions were germinated in the presence of trifluralin and malathion or NBD-Cl to assess the involvement of P450s and GSTs, respectively, in the observed trifluralin resistance. There was a significant difference (*p* < 0.001) between accessions using trifluralin. The root lengths of the resistant (4.4 ± 1.2 cm) accession on agar plates with 1 µM trifluralin were not different from the control (5.2 ± 0.9 cm), whereas roots of the susceptible (0.7 ± 0.2 cm) accession were swollen and reduced in size (Figure 2A,B).

The combination of trifluralin + malathion did not significantly (*p* ≥ 0.05) reduce root length of the resistant (3.4 ± 1.6 cm) accession compared to trifluralin alone. Despite this finding, the root length of some seedlings appeared to be affected by the application of trifluralin + malathion, suggesting a differential response of P450s (Figure 2C). Thus, under continuous selection pressure and with no weed management diversity strategies, these seedlings would contribute to P450-metabolism-based resistance, and it would not be rare to have both P450 and GST enzymes acting together in other trifluralin-resistant accessions (Figure 2C,D). In fact, in a related *Amaranthus tuberculatus* (Moq.) Sauer accession resistant to *S*-metolachlor, both P450s and GSTs were reported to contribute to resistance [40]. Finally, root lengths of the resistant (2.1 ± 0.8 cm) accession when treated with trifluralin + NBD-Cl were significantly less than when treated with trifluralin alone (*p* < 0.001).

Results displayed a variability in root length within the resistant accession. For instance, there were some seedlings that were more affected by trifluralin compared to the other resistant seedlings (Figure 2B). In the plates treated with either trifluralin + malathion or trifluralin + NBD-Cl, there were some resistant seedlings that did not show similar root length reduction compared to the other resistant seedlings (Figure 2C,D). A feasible explanation of previous observations could be the presence of polygenic resistance, which usually involves a differential tolerance response. On the other hand, it has been demonstrated that trifluralin resistance is a recessive trait. In studies carried out with *S. italica*, trifluralin resistance was demonstrated to be recessive with no monogenic segregation [41]. In addition, inheritance studies in *L. rigidum* and *S. viridis* demonstrated that trifluralin resistance is monogenic and recessive [42,43], but at lower trifluralin rates polygenic resistance would be involved [41,42].

Results obtained in this research demonstrate the likely involvement of GSTs in trifluralin resistance (Figure 2D). Our findings agree with those found in a selected pendimethalin-resistant *Alopecurus myosuroides* Huds accession, in which it was demonstrated that nontarget site resistance was attributed to enhanced metabolism. In addition, members of the CYP81A family, together with GSTs from tau and phi classes, were reported to be involved in the nontarget site resistance mechanism [44].

Cytochrome P450 monooxygenase, glycosyl transferase, and glutathione *S*-transferase have been described as playing an important role in herbicide metabolic resistance [45]. Overexpression of some of these enzymes is known to confer resistance to multiple herbicide chemistry in some weeds [7,46]. Overexpression of *CYP81A12* and *CYP81A21* were reported as the resistance mechanism in an acetolactate synthase (ALS) -resistant *Echinochloa phyllopogon* (Stapf) Koso-Pol. accession [47]. Furthermore, it has been shown that CYP81As can confer resistance to acetyl CoA carboxylase (ACCase)-inhibiting herbicides [48]. In *Lolium* spp., participation of P450s, GSTs and other genes on herbicide detoxification have been reported [49,50,51]. In *A. palmeri* accessions resistant to the herbicide *S*-metolachlor, resistance was attributed to GSTs [10,52].

### 3.3. Tubulin Gene Sequencing

SNPs in the target site of trifluralin-resistant grass accessions have been associated with trifluralin resistance [30]. Such SNPs can be found in both *α* and *β*-*tubulin* genes; therefore, we explored both genes to have a better understanding of trifluralin resistance. Sequences obtained and their predictive protein were searched using the Basic Local Alignment Search Tool (BLAST) available at https://blast.ncbi.nlm.nih.gov/Blast.cgi (accessed on 24 May 2021) for nucleotide and protein, respectively.

**Figure 3:**
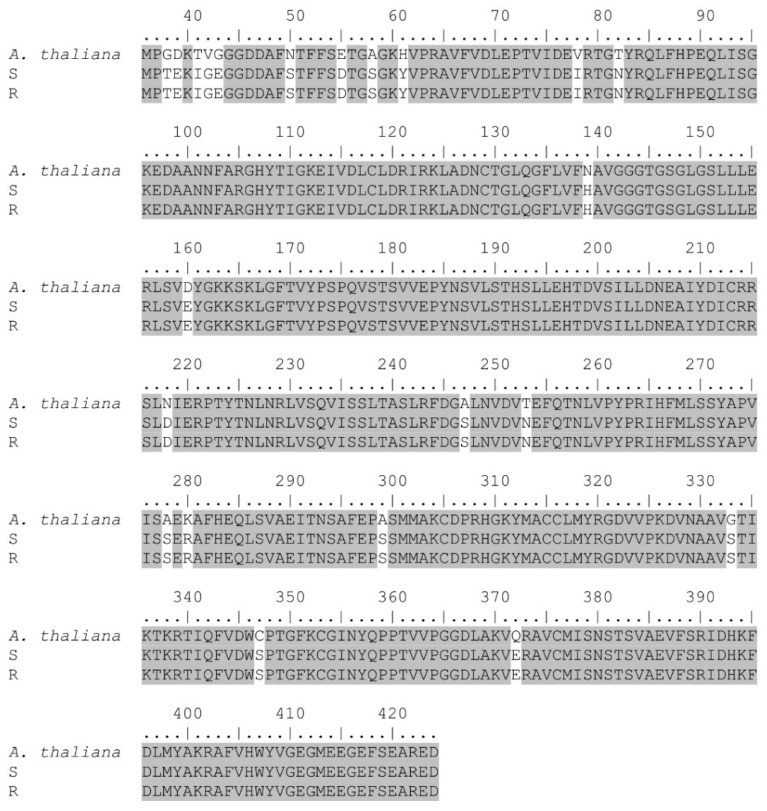
Partial protein sequence alignment of *α*-*tubulin* gene of trifluralin-susceptible (S) and resistant (R) *A. palmeri* accessions. Sequences were aligned to that of the *A. thaliana* (AT4G14960) *α*-*tubulin* gene. Highlighted color indicates homology among the different sequences.

Results demonstrated a high homology (88%) with *Chenopodium quinoa* Willd (GenBank accession: XM_021878117.1) and *β vulgaris* L. (GenBank accession: XM_010697176.2) *α*-*tubulin* sequences with an *E*-value (*Expect* value) of 0.0. A predictive protein search displayed 94% homology with *Spinacia oleracea* L. (GenBank accession: XP_021860086.1) and *C. quinoa* (GenBank accession: XP_021733809.1) *α*-*tubulin* protein sequences with 0.0 *E*-values (Figure 3).

**Figure 4:**
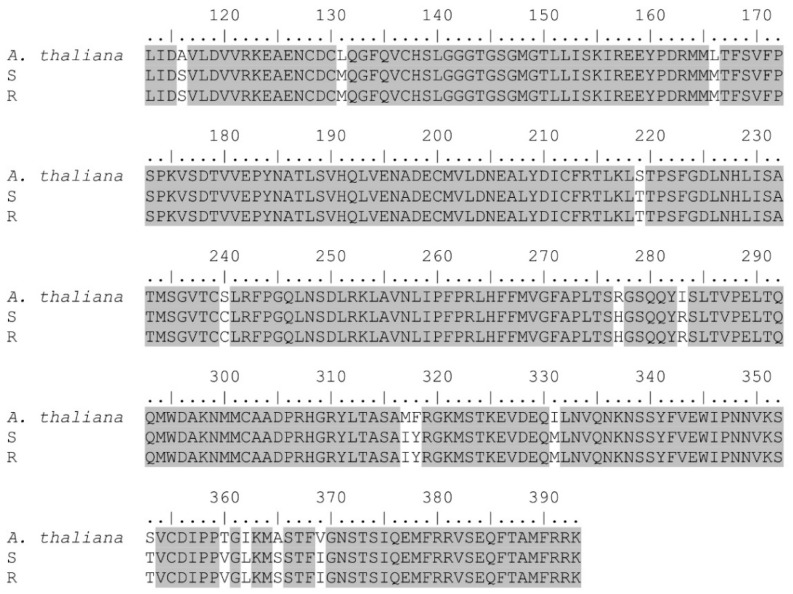
Partial protein sequence alignment of *β*-*tubulin* gene of trifluralin-susceptible (S) and resistant (R) *A. palmeri* accessions. Sequences were aligned to that of the *A. thaliana* (AT1G75780) *β*-*tubulin* gene. Highlighted color indicates homology among the different sequences.

In the same manner, results of *β*-*tubulin* demonstrated 88% homology with *B*. *vulgaris* (GenBank accession: XM_010687347.2), *C*. *quinoa* (GenBank accession: XM_021859747.1), and *S*. *oleracea* (GenBank accession: XM_022005515.1) *β*-*tubulin* sequences displaying *E*-values of 0.0. In addition, predictive proteins showed 97% homology with *Arabidopsis thaliana* (L.) Heynh. (GenBank accession: P29515.1) and *Lupinus albus* L. (GenBank accession: Q40106.1) *β*-*tubulin* protein sequences (*E*-values = 0.0.) (Figure 4).

Amino acid comparison between susceptible and resistant accessions showed similar protein sequences in both *α* and *β*-*tubulin* genes, indicating that a target-site mutation is not involved in the observed trifluralin resistance (Figure 3 and Figure 4). Despite this, we observed different SNPs in both the *α* and *β*-*tubulin* genes, and all of them were silent mutations. In trifluralin-resistant grass accessions, different mutations (mainly in *α*-*tubulin*) have been reported in contributing to resistance [15,20,22,23,24,30], but in this study with *A. palmeri* we did not observe any amino acid changes in the resistant accession. This discrepancy can be attributed to the weak trifluralin-resistance observed (RF = 2.6, see dose-response Section) and that the resistant accession can cope with trifluralin in other ways, such as GST or even P450-metabolism at a lower extent as demonstrated in this work. In trifluralin-resistant *L. rigidum* accessions with different resistance levels (e.g., ≤ 15-fold based on LD_50_), a target-site mutation was not reported in all the resistant-sequenced plants. Thus, Val-202-Phe and Thr-239-Ile in the *α*-*tubulin* gene were detected in only four out of 10 and seven out of 10 plants, respectively; no target-site mutation was reported in another trifluralin-resistant accession, suggesting that different resistance mechanisms could be involved within a population [24].

### 3.4. Tubulin Gene Copy Number Estimation

**Figure 5:**
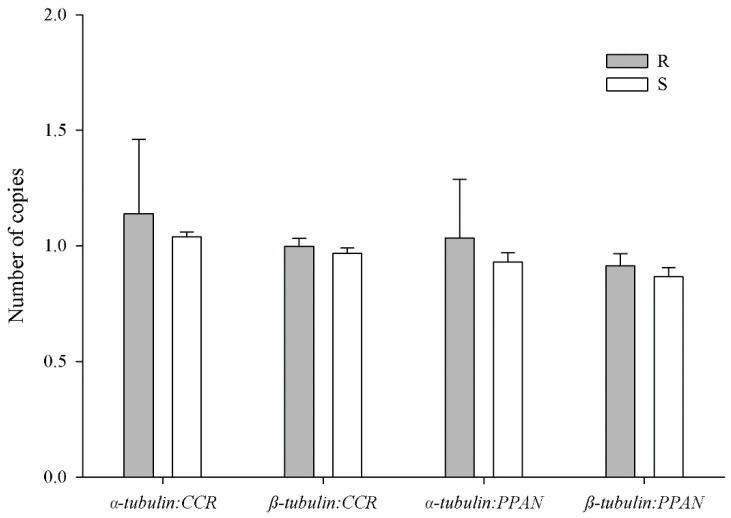
Gene copy number estimation of the *α* and *β*-*tubulin* genes relative to *CCR* and *PPAN* reference genes in trifluralin-resistant (R) and susceptible (S) accessions. Bars ± standard deviation of the mean (*n* = 4). A Student’s *t*-test analysis was performed to assess significant differences between accessions.

In this study, we estimated the *α* and *β*-*tubulin* gene abundance in trifluralin-resistant and susceptible *A. palmeri* accessions relative to *CCR* and *PPAN* genes. The rationale in using these genes instead of *ALS*, a common reference gene, is that the latter was found to be unstable in *Alopecurus aequalis* Sobol. [53]. Thus, we used *CCR* and added *PPAN* as a second reference gene as it has been suggested that *a priori* both are reported as a single gene copy [33,34,35,53]. Other genes such as *β*-*tubulin*, *carbamoyl phosphate synthetase*, and *RNA dead box helicase* have been used to estimate additional gene copy numbers in 4-hydroxyphenylpyruvate dioxygenase (HPPD)-resistant and glufosinate-tolerant *A. palmeri* accessions [11,39,54]. In this study, similar *α* and *β*-*tubulin* gene copies were observed in both resistant and susceptible *A. palmeri* accessions, indicating that amplification of the *α* and *β*-*tubulin* genes are not involved in the observed trifluralin resistance (Figure 5). As both *α* and *β*-*tubulin* genes are in equilibrium to maintain cell viability, resistance mechanisms to trifluralin such as gene amplification or overexpression would be unlikely unless both genes are involved [30].

Our results are similar to those reported in glufosinate-tolerant and HPPD-resistant *A. palmeri* accessions, where gene amplification of the target-site gene was not correlated with the resistance mechanism [11,39,54]. Nonetheless, gene amplification has been described in other herbicide-resistant weed accessions, mainly to glyphosate. In *A. palmeri*, *Kochia scoparia* (L.) Schrad., and different grass accessions, amplification of the target gene was involved in the resistance mechanism [34,37,55,56,57,58,59,60,61].

## 4. Conclusions

In this research, we explored the basis of resistance in a trifluralin-resistant *A. palmeri* accession. In both the *α* and *β*-*tubulin* genes of the resistant accession used in this study, SNPs were not found at the positions where mutations have been described before in conferring trifluralin resistance. While unlikely, it is acknowledged that in the rest of the amino acid sequence not obtained there could potentially be mutations that have yet to be associated with trifluralin resistance. In addition, gene amplification of the *α* and *β*-*tubulin* genes is not involved in the resistance mechanism. Nonetheless, our findings support the involvement of GSTs as contributing to trifluralin resistance in this *A. palmeri* accession. Research is required to understand the role of all GSTs, and other metabolism-related genes, that may control metabolism resistance. For that reason, future efforts will focus on understanding which GSTs are upregulated and responsible for endowing resistance to this trifluralin accession. Furthermore, the response of this accession to other herbicides for which GST-mediated resistance is known will be evaluated.

## Figures and Tables

**Table 1:** Primer sets used to calculate the relative *α* and *β*-*tubulin* gene copy number by qPCR in trifluralin-resistant and susceptible *A. palmeri* accessions.

Gene ^1^	Sequence (5′ → 3′) ^2^	Amplicon (bp)	Slope	Efficiency (%) ^3^
*α*-*tubulin*	F GAGAAGGTGGAGACGATGCAT R AATTCCCGGTCCGAATTTCGT	123	−3.352	98.8
*β*-*tubulin*	F GCTGTCTTAGGTTCCCAGGTC R GCCATGAGAGGTTAGAGGAGC	119	−3.421	96.0
*CCR*	F CGACGGAAAATAGCAACAAAGTG R GTCTTTGACGGTGGCGTTAAC	116	−3.355	98.6
*PPAN*	F TGCTCCATTTTTGAGGGTTGC R GACATCGAGGCCTCAACTGTG	113	−3.3033	100.8

^1^*α*-*tubulin*, *α*-*tubulin*; *β*-*tubulin*, *β*-*tubulin*; *CCR*, *Cinnamoyl*-*CoA reductase*; *PPAN*, *peter Pan*-*like*. ^2^ F, forward; R, reverse. ^3^ Efficiency was calculated as E = [10^(−1/slope)^ − 1] × 100.

## Data Availability

Not available.
